# Site directed biotinylation of filamentous phage structural proteins

**DOI:** 10.1186/1743-422X-8-495

**Published:** 2011-11-01

**Authors:** Larisa Smelyanski, Jonathan M Gershoni

**Affiliations:** 1 Department of Cell Research and Immunology, George S. Wise Faculty of Life Sciences, Tel Aviv University, Tel Aviv, 69978 Israel

**Keywords:** filamentous bacteriophage, biotinylation, phage display, combinatorial libraries

## Abstract

Filamentous bacteriophages have been used in numerous applications for the display of antibodies and random peptide libraries. Here we describe the introduction of a 13 amino acid sequence LASIFEAQKIEWR (designated BT, which is biotinylated *in vivo *by *E. coli*) into the N termini of four of the five structural proteins of the filamentous bacteriophage fd (Proteins 3, 7, 8 and 9). The *in vivo *and *in vitro *biotinylation of the various phages were compared. The production of multifunctional phages and their application as affinity reagents are demonstrated.

## Background

Biotinylation of proteins, nucleic acids, lipids and sugars is without a doubt one of the most fundamental tools of modern cell biology and biotechnology. This is due to the scarcity of naturally biotinylated proteins (< 5 per organism) [1-3], the chemical flexibility by which biotin can be covalently conjugated to specific moieties of biopolymers and organic ligands, and the exceptional high affinity binding between avidin/streptavidin and biotin (K_D _= 10^-14^M) [4]. Hence, biotinylation provides the means for extremely efficient affinity labeling and purification of macromolecules, effective monitoring or tracking sub-cellular events, as well as the production of a wide range of diagnostics, to mention only a few of the areas where the Avidin/Biotin Complex ("ABC") is now employed routinely [5,6].

Biotin (vitamin H) naturally exists as an obligatory co-factor of carboxylase enzymes and functions as a carboxyl-carrier. These enzymes are ubiquitous, existing in all organisms ranging from microbes to mammals [1-3]. In *E. coli *biotin carboxyl carrier protein (BCCP) is the only protein that is biotinylated and is one of four subunits of the enzyme acetyl CoA carboxylase which is functional in fatty acid biosynthesis. BCCP is biotinylated on the epsilon amine of lysine 122 via an amide bond formed at the expense of ATP [7,8], a reaction that is catalyzed by the biotin holoenzyme synthetase (BHS the product of the *BirA *gene) [9]. The intact BCCP is 156 amino acids long (16.7 kDa) however, the actual cues for biotinylation are contained within only the last carboxy terminal 66-75 amino acid residues of the protein. Thus it has been illustrated that fusion proteins expressing the biotinylation sequence are efficiently biotinylated *in vivo *by *E. coli *or *in vitro *reaction-mixtures containing BHS [10-12]. Attempts to dramatically trim the size of the peptide while maintaining its capacity for biotinylation *in vivo *have failed leading to the conclusion that the enzyme requires a distinct conformation around its Lys 122 (AMKM) and also depends on additional contacts provided by other residues contained within the minimal sequence of 30 amino acids before and after the critical Lysine. Nonetheless, Schatz [13] has demonstrated that a 13-15aa peptidomimetic of the lysine-containing turn can be produced that is efficiently recognized by BHS and can easily be used as a biotinylation tag (BT) either N' terminal or C' terminal to recombinant fusion proteins expressed in *E. coli *(available now as a commercial biotinylation tag coined "Avi-Tag", GeneCopoeia, Inc., Rockville).

In this study we report the successful expression of the BT at the N' termini of four of the five structural proteins of the fd filamentous bacteriophage (Proteins 3, 7, 8, and 9). Each protein can be biotinylated *in vivo *generating site-specifically biotinylated infectious filamentous phage. The biotinylation of phages and their potential applications are discussed.

## Methods

### Construction of BT fused phage proteins

All phages were produced using the fth1 vector [14] which were grown, harvested and analyzed by dot blot and ELISA using standard protocols as previously described in Tarnovitsky *et al*. [15]. Oligonucleotides corresponding to the BT were introduced at the N' terminal aspects of Proteins 3, 8, 7 and 9 as follows:

#### Protein 3

**Figure 1:**
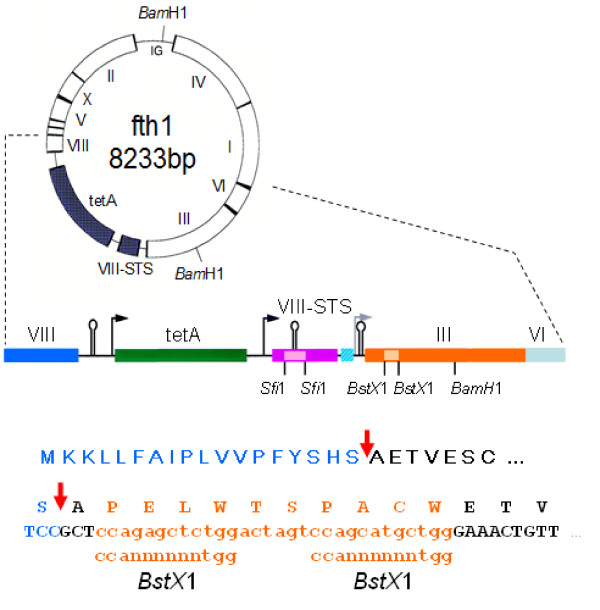
**Modification of fth1 vector for expression of peptides at the N-terminus of Protein 3**. The fth1 vector containing a second recombinant *protein 8 *gene was further modified at the native *protein 3 *gene. Two asymmetric *BstX*1 restriction sites were introduced between the codons of Ala1 and Glu2 of Protein 3. The BT was subsequently cloned into the BstX1 cloning cassette.

The modified fth1 vector shown in Figure 1 contains two asymmetric *BstX*1 sites immediately following the GCT alanine first codon of the N-terminus of mature Protein 3. The following oligonucleotides coding for the BT flanked with compatible overhangs -

p3BT-sense 5'-CTGGCTAGCGTCTATCTTCGAGGCCCAAAAGATCGAGTGGCGACCAGACGT-3'

p3BT-antisense 5'-CTGGTCGCCACTCGATCTTTTGGGCCTCGAAGATAGACGCTAGCCAGAGCT-3'

were cloned into *BstX*1 digested vector. DH5alpha cells were transformed and BT containing clones were isolated and confirmed for the correct sequence as well as for BT expression as described in the text.

#### Protein 8

Phages containing BT fused to Protein 8 were generated using two different approaches. The following oligonucleotides coding for BT flanked with compatible *Sfi*1 overhangs were used in both systems

p8BT-sense 5'-ATCGCTAGCCTCTATCTTCGAGGCCCAAAAGATCGAGTGGCGATCTG-3'

p8BT antisense 5'-ATCGCCACTCGATCTTTTGGGCCTCGAAGATAGAGGCTAGCGATCGT-3'

Direct cloning of the BT into fth1 was performed using fth1 restrict digested with *Sfi*1 and transformation of DH5alpha cells. Alternatively, the gene for recombinant Protein 8 derived from fth1 and containing the *Sfi*1 cloning cassette was cloned into pUC18 expression vector (GeneScript USA Inc., Piscataway NJ). This vector was digested with *Sfi*1 into which the BT insert was subsequently cloned. Similarly this modified pUC18 vector was used to generate a recombinant Protein 8 expressing the mAb CG10 binding peptide: CAKEGDLNKYKPWC [16]. In both cases DH5alpha cells were transformed with the pUC18 expression vectors and infected with various phages. This led to the production of phages that could assemble multiple Protein 8 units displaying a variety of different peptides.

#### Proteins 7 and 9

The construction of BT containing Protein 7 and Protein 9 was performed using "gene SOEing" Overlapping PCR as previously described in the construction of the fth1 vector [14]. It is noteworthy that the termination codon of Protein 7 overlaps with the initiation codon of Protein 9 in an alternative reading frame. Hence the integrity of both must be maintained during the construction of the fusion proteins. Oligonucleotides corresponding to the BT described above were introduced into the 5' aspect of the Protein 7 and Protein 9 genes immediately after the first ATG codon.

#### Biotinylation *in vivo *and *in vitro*

**Figure 2:**
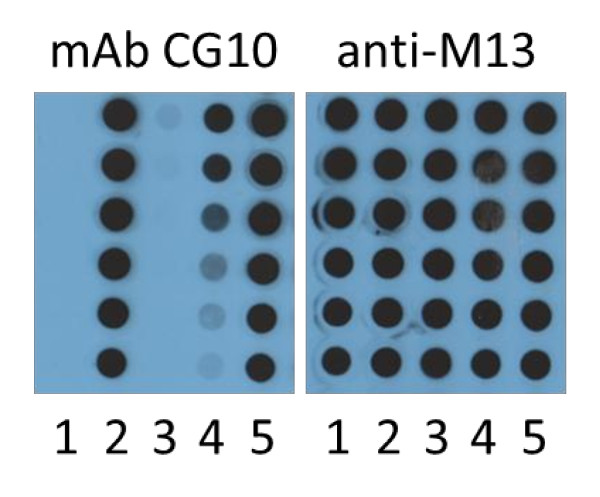
**The effect of *lac-i *inactivation**. The peptide recognized by the mAb CG10 was expressed on Protein 8 of fth1 phage. Protein 3 of the same phage contained the BT. As is illustrated in the dot-blot above, mAb CG10 binds 2X serial dilutions of the phages (column 2) as opposed to the total lack of binding of the control phage fth1 (column 1). Expression of the phages in DH5alpha cells transformed with pBIRAcm dramatically reduces the expression of the CG10 epitope (column 3). Addition of IPTG to the cells induces the expression of the recombinant Protein 8 (column 4). Inactivation of the *lac-i *gene by digestion with *Apa*1 and self-ligation dramatically improves the expression of the recombinant Protein 8 without the need of IPTG (column 5). Reaction of a duplicate filter with anti-M13 shows that the concentration of phages for the 5 samples is the same. The *in vivo *biotinylation of Protein 3 was measured by ELISA and samples 3-5 gave equal signals (1.8-2.0 OD) as the promoter of Protein 3 is not repressed by *lac-i *(not shown).

The biotinylation of the recombinant phage proteins + BT occurs in *E. coli *via endogenous BHS. However, addition of 100 μM d-biotin (Sigma B-4501) to the growth medium enhances the level of biotinylation. Further substantial improvement can be achieved by transforming the bacteria with the pBirAcm plasmid (GeneCopoeia, Inc., Rockville) which contains the *BirA *gene under the control of the tac promoter. This plasmid also contains the *lac-i *gene. As a result the expression of recombinant Protein 8 of the fth1 system is markedly repressed in pBirAcm transformed cells as it too is driven by tac promoter. The repression can be overcome to some degree by isopropyl β-D-1-thiogalactopyranoside (IPTG) induction. However, marked improvement is observed if the *lac-i *gene is simply inactivated (see Figure 2). Inactivation of the *lac-i *gene was accomplished by cleavage of the pBirAcm plasmid with *Apa*1 which produces 3' overhangs. These were polished to generate blunt ends using DNA Polymerase 1 large fragment (Klenow). Such treated vectors were then self-ligated which leads to a truncated inactive *lac-i *gene product.

In vitro reactions were conducted as per the manufacturer's protocol using the commercial Biotin Protein Ligase (EC 6.3.4.15, GeneCopoeia, Inc., Rockville).

#### Solid phase immunoassays

Generally, ELISA and dot blots were performed as previously described [15]. Briefly, biotinylated bovine serum albumin (Sigma, A8549) was plated into wells of 96 well plates in Tris buffered saline (TBS, pH7.5), washed, quenched with TBS/milk solution and incubated with streptavidin (10 μg/ml in TBS) and washed. The wells were then incubated with the various phages as indicated in the text and figures, washed and reacted with rabbit anti-M13 followed by goat anti rabbit-IgG horseradish peroxidase conjugate. The wells were developed with 3,3',5,5'-tetramethylbenzidine substrate solution and read at 650 nm in a BioTek plate reader. Dot blots were prepared using nitrocellulose membrane filters (BA 85 Whatman) and a 96 well vacuum manifold (Whatman). Filters were quenched with TBS/milk and reacted with antibodies as indicated in the text and developed for enhanced chemiluminescence (ECL).

#### Affinity depletion and purification of antibodies from polyclonal serum

Streptavidin magnetic beads (20 μl in 0.5 ml solution) were incubated an hour at room temperature with 10^10 ^phages expressing the HIV-1 gp41 pentameric loop epitope (603-CSGKLIC-609) on Protein 8 and the BT on Protein 7, or not. Serum from an HIV infected individual (patient #I9809 [17]) was incubated with the beads for one hour at room temperature. The bound antibodies were eluted from the magnetic beads after TBS washes and acidification with elution buffer (glycine-HCl pH 2.2+1% BSA) as previously described [17]. The void non-bound antibodies were collected as well. The different fractions were analyzed by dot blot. For comparison phages expressing a second epitope (CWGGTNWGQTPIVC, [17]) were also used.

## Results

Filamentous bacteriophages have been used extensively as expression systems for the production of combinatorial phage-display peptide [18,19] and antibody libraries [20,21]. Although all five structural proteins have been demonstrated as compatible for expression of fusion proteins [22], two phage proteins have been found to be particularly efficient.

### Construction of site biotinylated phage proteins

#### Protein 3

**Figure 3:**
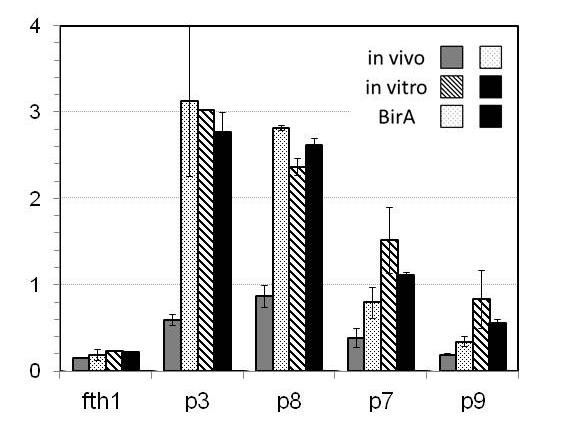
**Biotinylation *in vivo *and *in vitro *of phage proteins 3, 7, 8 and 9**. The BT was introduced into the phage proteins 3, 7, 8 and 9 and compared with fth1 phage as indicated. The phages were tested by quantitative ELISA for the presence of biotin with (stipple and black) or without (grey and hatched) *lac-i *modified pBIRAcm for *in vivo *biotinylation (grey and stipple) compared with subsequent *in vitro *biotinylation (hatched and black).

Protein 3 is a multi-domain protein of 406aa present in 3-5 copies at the distal tip of the phage [23]. It is easily compatible with the expression of foreign peptides as large as a few hundred amino acids long and as such is typically used in the production of antibody libraries [20,21]. Foreign DNA inserts can be introduced into a number of sites in this protein. As is shown in Figure 1, using over-lapping PCR we have modified the fth1 expression vector and introduced a 30 bp insert containing a pair of *BstX*1sites between positions 1 and 2 of the native protein. In such an instance all the Protein 3's of the phage are genetically modified. In order to first confirm that this new cloning site is compatible with functional expression, the amino acid sequence AGFAIL which corresponds to the epitope of the murine monoclonal antibody GV4H3 [24] was introduced into the modified *p3 *gene. The specifically modified phages acquire specific recognition by the GV4H3 mAb as expected (not shown). Replacing the GV4H3 epitope with the 13 amino acid biotinylation tag (BT = LASIFEAQKIEWR, [13]) generated a phage that incorporated biotin that was confirmed by streptavidin binding (Figure 3).

#### Protein 8

Protein 8 encapsidates the entire length of the viral ssDNA. Some 2700 copies of Protein 8 form a helical tube with a pitch of 5 protein copies per turn. The protein is 50 residues long of which the greater part forms an alpha helix that can close-pack against neighboring Protein 8's, where the carboxy termini interact electrostatically via a cluster of four lysine residues with the DNA while the N termini are free and amenable to molecular manipulation [25]. Genetic alteration of the phages' single *p8 *gene leads to a phage that becomes homogenously modified along its entire shaft where all 2700 copies contain the foreign insert. It turns out, however, that inserts exceeding 6-8 residues in length interfere with the packing of the Protein 8 into the growing filament of the phage [18,26]. Expression of longer Protein 8 fusions is possible however. This can be achieved when two *p8 *genes are functional; one expressing the wild type Protein 8 and the other the recombinant Protein 8 containing the foreign peptide - ultimately leading to the production of a "mosaic phage" where most of its Protein 8 is wild type, interspersed with copies of recombinant Protein 8 [27]. The fth1 vector developed in our lab is a "type 88" [14] vector which downstream to the natural wild type *p8 *gene, contains a second recombinant *p8 *gene modified by the introduction of a pair of *Sfi*I cloning sites in place of the native Asp residue 4 of the natural protein. This vector has been used extensively to produce phage display peptide libraries that express inserts ranging from 3-50 residues long.

For the incorporation of BT into Protein 8 we adopted two approaches. As is shown in Figure 3 a DNA insert corresponding to the BT can be introduced directly into the *Sfi*I cloning sites rendering the production of Protein 8 biotinylated phages.

**Figure 4:**
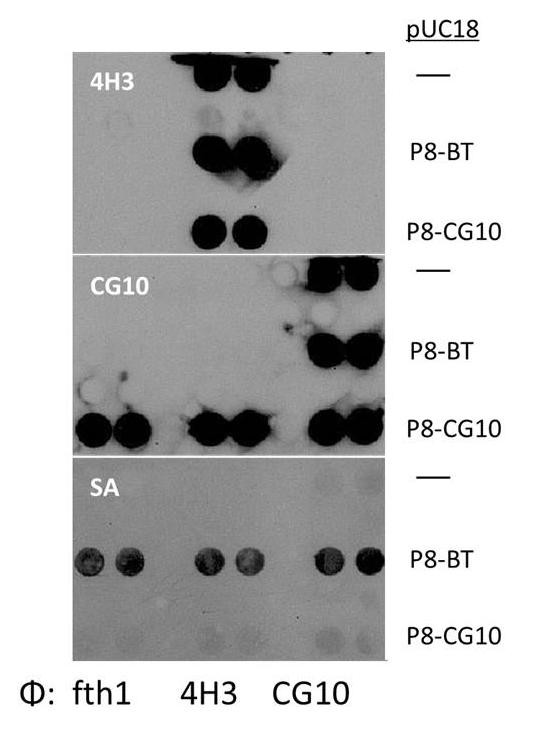
**Production and biotinylation of multi-functional phages**. Phages were produced either without any insert (fth1), or containing epitopes for mAbs 4H3 or CG10, both antibodies directed against HIV gp120. The three phages were then used to infect *E. coli *bacteria that had been transformed with either pUC18 expressing a Protein 8 fusion with the CG10 epitope or with BT as indicated. Three identical dot-blots of the phages generated from these transformed cells were probed with mAbs 4H3, CG10 or with Streptavidin-HRP (SA). All the phages derived from the BT-transformed bacteria express biotinylated Protein 8 as well as the other corresponding markers, thus illustrating the possibility for multi-functionality of the phages.

An alternative approach for expression of multiple *types *of Protein 8 in a single phage is also possible. For this we constructed a pUC18 plasmid for the expression of an independent recombinant Protein 8 in a bacterium infected with the fth1-phage. As is shown in Figure 4, we have performed secondary modification of three distinct phages: (i) fth1 phage that expresses only wild type Protein 8 (as no insert is introduced in the second recombinant *p8 *gene of fth1), (ii) a "4H3" phage where the AGFAIL peptide corresponding to the GV4H3 mAb described above has been cloned into the *Sfi*I sites of the second *p8 *gene of the fth1vector, and finally (iii) the "CG10" phage that expresses via the *Sfi*I cloning sites a peptidomimetic of the conformational epitope corresponding to the CG10 mAb [16]. Each of these phages was then used to infect *E. coli *that had previously been transformed with the pUC18 plasmids containing a Protein 8 fusion in which either the BT or the CG10 peptidomimetic are expressed. As is seen, all three phage-types become detectable with HRP conjugated streptavidin when they are grown in pUC18-BT transformed bacteria. This system illustrates that one can easily introduce into a single phage a diversity of Protein 8 recombinants. It also proves the possibility of simple biotinylation of pre-existing phages expressing desired peptides or antibodies by simply culturing them in the pUC18-BT transformed bacteria.

#### Proteins 7 and 9

There has been little documented experience in modification of Proteins 6, 7 and 9. Protein 6 packs with Protein 3 at the distal tip of the phage and has been used for carboxy-terminal expression [28]. At the other end of the phage, Proteins 7 and 9 are involved in recognition of the DNA packaging signal of the phage (a short 78 base hairpin in the ssDNA [29]) and are thought to initiate the extrusion of the phage particles thus forming the proximal tip of the mature phage. The compatibility of Protein 7 or Protein 9 with the expression of foreign peptides as fusions has been somewhat unclear. Endemann *et. al*. initially reported that only Protein 9 was accessible to the surface of the phage yet could not be used for expression [30]. Since then Gao *et. al*. illustrated that both Protein 7 and Protein 9 tolerate N-terminal manipulation (as opposed to lack of compatibility of the C termini of both proteins) yet in order to obtain expression it appeared that a phagemid system was required ensuring that at least some wild type Protein 7 and Protein 9 were co-expressed in the phages [31]. Maintaining these conditions, Gao expressed VL domains of a number of antibodies in Protein 7 while simultaneously expressing the cognate VH of each mAb on Protein 9 thus realizing functional specific antigen recognition illustrating that both phage proteins could be employed for functional N-terminal fusions. As is depicted in Figure 3 we constructed modified Protein 7 and Protein 9 as N-terminal fusions of BT. In both cases we were able to detect streptavidin binding to recombinant phages. This indicates, therefore, that both proteins are compatible with fusion protein expression without the need of a phagemid-derived second wild type protein. Modification of Protein 7 and Protein 9 in this case does, however, reduce the total amount of phages produced resulting in titers markedly lower than would otherwise be expected. One might question however, whether or not the modified Protein 7 or Protein 9 interfere with the function of these proteins in infectivity. Therefore, we tested the ability of the modified phages to infect DH5alpha cells. In these experiments it was found that bacteria incubated with phages containing recombinant Protein 7 or Protein 9 could infect the cells as indicated by acquisition of resistance to tetracycline as well as continue to produce recombinant progeny illustrating that the modified proteins are able to participate in the extrusion of functional phages.

### *In vivo *vs. *in vitro *phage biotinylation

The four phage protein constructs described above illustrate that the BT can be incorporated functionally into the proteins and produce biotin-containing phages which are extruded into the media. The question is whether the majority of BT's are actually biotinylated *in vivo *or rather are expressed but missed by the cytoplasmic enzymatic machinery? Should the latter be the case, one could expect an increase of biotinylation per phage in subsequent *in vitro *biotinylation reactions. Hence the following experiments were conducted.

First, the effect of adding biotin to the culture medium was tested and found that addition of 100 μM of biotin improved the level of *in vivo *biotinylation (not shown). However, the most dramatic improvement was seen when bacteria were co-transformed with a plasmid containing the *BirA *gene (see Figure 3). Thus five different phages were compared for biotinylation: phages containing N-terminal BT for Proteins 3, 8, 7 and 9 respectively compared to the fth1 phage as a negative control. The level of biotinylation, as monitored by quantitative ELISA using Streptavidin-HRP as the probe, was measured for biotinylation *in vivo *in the presence or absence of the *BirA *plasmid. The harvested phages were then subjected to *in vitro *biotinylation. As can be seen for Proteins 3 and 8 *in vivo *biotinylation was markedly enhanced in the bacteria co-transformed with the *BirA *containing plasmid. The subsequent *in vitro *reactions did not however, improve the level of biotinylation substantially. This most probably indicates that for these two proteins the N-terminal BT is accessible in the cytoplasm of the bacterium. Thus increasing the level of *BirA *improves the efficiency of biotinylation and for the most part the majority of BT's become pre-tagged before the phage is extruded. This does not seem to be the case for Proteins 7 and 9. Here *in vitro *biotinylation improves the level of biotin incorporation. Curious however is the observation that elevating cellular *BirA *tends to reduce the availability of sites for subsequent *in vitro *biotinylation. This could indicate that biotin-tagging of these cellular proteins interferes to some degree with their membrane transport, assembly or phage extrusion.

### Affinity depletion and purification of antibodies using biotinylated phages

#### Affinity depletion of dominating antibodies from polyclonal serum

**Figure 5:**
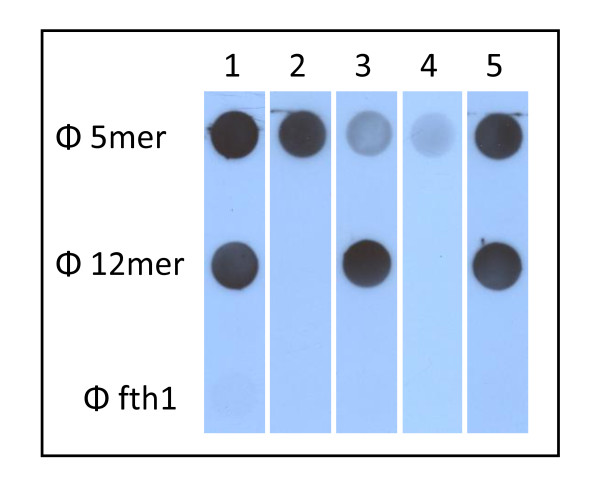
**Selective affinity depletion of serum using biotinylated phages**. Phages "12mer" and "5mer" express two epitopes of HIV (12mer: CWGGTNWGDTPIVC, 5mer: CSGKLIC). The total activity of HIV+ polyclonal serum is shown in strip #1. The serum was applied to 5mer-phages biotinylated on Protein 7 and bound to streptavidin magnetic beads. Strip #2 illustrates the affinity purified antibodies specific for the 5mer eluted off the column. Strip #3 shows the depleted serum after passing over the 5mer column. As a control the columns were prepared using the same 5mer phages but not biotinylated at all. Strip #4 shows that in this situation practically no antibody can be eluted off the column and all the activity passes through the column as is shown in strip #5.

HIV-1 infected individuals tend to mount a strong antibody response towards the gp41 pentameric loop (residues 603-CSGKLIC-609) [17]. Biopanning polyclonal serum typically generates numerous phages that represent this dominating epitope often to the extent that other activities are overshadowed. Hence it becomes desirable to reduce the dominance of the anti-pentameric loop response by selective depletion of those antibodies specific for this epitope. For this, a phage displaying the pentameric loop on Protein 8 and biotinylated on Protein 7 was used as an affinity reagent. Polyclonal serum was applied to the biotinylated phages bound to streptavidin-magnetic beads. As is illustrated in Figure 5 the serum that flows through the beads is markedly depleted of antibodies specific for the pentameric loop. The specific antibody can then be eluted off the column. As expected affinity depletion or purification is not achieved using phages that express the pentameric loop but are not biotinylated.

## Discussion

Here we have reported the site specific biotinylation of four of the five structural proteins of the fd filamentous bacteriophage. Biotinylation of bacteriophages has been reported in the past by others. The majority of these studies have involved the chemical modification of mature infectious phages by standard means such as the use of the N-hydroxy succinimide ester of biotin [32,33]. Whereas this is an efficient way to incorporate biotin onto phages, such chemical procedures do not afford the specificity or the control to attach the biotin to one antigen or other or restrict the modification to a discrete lysine residue. Moreover, the chemical biotinylation could incorporate biotin to critical lysines or other selected residues and lead to interference with phage function (note for example that the pentameric loop of gp41 contains a lysine residue). *In vivo *biotinylation of phage capsid proteins has been demonstrated for phages such as lambda whose assembly is cytoplasmic and does not require membrane transport of biotinylated proteins as is the case for filamentous phages (see below). Ansuini *et. al*. exploit this fact in an elegant report in which the BT serves as a tag for functional open reading frame expression of cDNA libraries [34].

The biosynthetic pathway for filamentous phage extrusion is exceptionally interesting as it is complicated [23,35,36]. Briefly, the phage genome encodes for 9 open reading frames plus two over-lapping reading frames giving 11 proteins. Of these, five are structural proteins comprising the proximal tip of the phage (Proteins 7 and 9), the shaft of the phage (Protein 8) and the distal tip (Proteins 3 and 6). At the initiation of assembly the phage structural proteins are all present as integral membrane proteins of the inner membrane with their N termini facing the periplasmic space. At first, the ssDNA to be packaged is encapsidated in cytoplasmic Protein 5 which has a distinct binding preference for single strand DNA [29]. Thus a short double stranded hairpin of the phage-DNA is devoid of Protein 5 and serves as a packaging signal recognized by the periplasmic membrane associated proteins, Proteins 9 and 7. Binding of these to the packaging signal initiates phage assembly. Now membrane associated Protein 8 moves laterally in towards the site of assembly sequentially replacing tiers of the Protein 5 subunits thus building the new capsid of the phage stepwise and extending outward into the medium [37]. When the DNA is fully encapsidated with Protein 8 subunits, Proteins 6 and 3 join the ensemble and effectively allow the fully extended phage to snip off into the medium without causing the bacterium to lyse [38]. The fact that all four of the proteins studied in this report can be detectably biotinylated would suggest that either biotinylation occurs in the periplasm or that cytoplasmically biotinylated protein can be translocated through the inner membrane.

Biotin holoenzyme synthetase (BHS), the *BirA *gene-product, is in fact a cytoplasmic enzyme [1,9]. Moreover, it has been demonstrated that cytoplasmically biotinylated proteins such as alkaline phosphatase or maltose binding protein can be transported into the periplasmic space [39,40]. This thus illustrates that the presence of biotin *per se *does not necessarily interfere with membrane transport in *E. coli*. Transport of proteins in *E. coli *is mediated by a number of different and distinct systems (e.g., SRP, Sec, and YidC transport systems [41,42]). Manipulating the transport system can have a profound effect on the efficacy of biotinylation where slowing the transport down tends to increase the yield of *in vivo *biotinylation of proteins destined to be transported into the periplasmic space [39,40]. Steiner *et. al*. illustrated that replacing the Sec translocation signals for co-translational SRP translocation signals improved dramatically the display of proteins on phage protein 3 [43].

Regardless the translocation system, all four phage proteins described here were found to be biotinylated *in vivo *and assembled into functional phages. However, cytoplasmic tagging of Proteins 7 and 9 did seem to impair the yield of phage production.

The existence of the biotinylated phage components may be useful for the study of phage biosynthesis and assembly and should afford opportunities in following the pathway of the proteins from ribosomal translation to membrane transport and assembly. One may also be able to exploit these constructs to follow the fate of the protein components of incoming infectious phages into the host bacterium.

The ability to efficiently biotinylate phages with precision should open new avenues for the biotechnological application of phages. *In vivo *biotinylation of phage Protein 3 has already been reported. Scholle *et. al*., for example, devised a unique system whereby a short random peptide library expressed on Protein 3 is followed by an AviTag sequence. The biotinylated phages are then captured on immobilized Streptavidin and reacted with proteases. Those phages that are released display peptides that are cleavable by the enzyme being investigated [44]. As shown here peptide specific phages can be produced carrying endogenous biotin moieties without the concern of effecting the properties of the affinity ligand and shown to enable antibody depletion or affinity purification. Certainly the same could be done using phages that express antibodies or receptor-specific ligands and thus generate easily obtained capture reagents for purification protocols. More intriguing could be the unique situation whereby the use of targeted *in vivo *biotinylation we can selectively tag one tip or other of the phage or phages with multiple functional moieties. Thus one can envision the oriented immobilization of phages to solid supports. Directional and orientated immobilization of phages could produce distinct optical signatures as opposed to random immobilization. This and other attributes of targeted biotinylation could afford new applications for phages where precise and regulated manipulation may be required.

## List of abbreviations

BCCP: biotin carboxyl carrier protein; BHS: biotin holoenzyme synthetase; BT: biotinylation tag; ECL: enhanced chemiluminescence; ELISA: enzyme-linked immunosorbent assay; HRP: horseradish peroxidase; Sec: secretory; SRP: signal recognition particle.

## Competing interests

The authors declare that they have no competing interests.

## Authors' contributions

Both authors contributed to the design and execution of the experiments described and read and approved the final manuscript.
